# The Overlooked Issue of Outpatient Combination Antibiotic Prescribing in Low- and Middle-Income Countries: An Example from Syria

**DOI:** 10.3390/antibiotics11010074

**Published:** 2022-01-09

**Authors:** Ana Tomas, Saleh Aljadeeah

**Affiliations:** 1 Department of Pharmacology, Toxicology and Clinical Pharmacology, Faculty of Medicine, University of Novi Sad, Hajduk Veljkova 3, 21000 Novi Sad, Serbia; ana.tomas@mf.uns.ac.rs; 2 Institute of Medical Management and Health Sciences, University of Bayreuth, Prieserstr 2, 95440 Bayreuth, Germany

**Keywords:** antibiotic prescription, antimicrobial resistance, antimicrobials, drug utilization research, dual antibiotic therapy, Syria

## Abstract

This study aimed to determine and describe the prevalence of combination antibiotics dispensed in outpatients with health insurance in Syria. Data on all dispensed medicines between June 2018 and May 2019 for 81,314 adults were obtained, and medicines belonging to the J01 group of the World Health Organization (WHO) anatomical therapeutic classification (ATC) were included in the analysis. Prescriptions were stratified according to the number of antibiotics, age, and sex. Antibiotic utilization was expressed as the number of prescriptions per 1000 persons per year. Out of 59,404 prescriptions for antibiotics, 14.98% contained antibiotic combinations, distributed to 22.49% of the patients. The prevalence of dispensing antibiotic combinations was higher in female patients (23.00%), and the youngest (18–30 years, 26.19%) and oldest age groups (>70 years, 25.19%). The antibiotics most commonly combined were co-amoxiclav, second- and third-generation cephalosporins, and macrolides. Over 60% of the combinations contained ceftriaxone alone or in combination with sulbactam. The present study shows an alarmingly widespread prescription of antibiotic combinations, posing a risk to global health by promoting resistance development.

## 1. Introduction

The rate of antimicrobial resistance (AMR) has grown substantially over the past two decades, posing a major threat to global health [1]. There are many drivers of AMR, including the use and misuse of antibiotics in human medicine [2]. The use of antibiotics is one of the most important modifiable risk factors for the development of AMR. Data on antibiotic consumption are scarcer for low- and middle-income countries ((LMICs); the low-income gross national income per capita is USD 1045 or less, and the lower–middle-income gross national income per capita is between USD 1046 and USD 4095, calculated using the World Bank Atlas method [3]) compared with high-income countries [4,5], while the available literature suggests that the AMR prevalence in LMICs is higher than reported in high-income countries [6]. The available data on the sale of antibiotics suggests a remarkable degree of variation in the quantity and patterns of antibiotic prescriptions across low-, middle-, and high-income countries [7]. There are several practical issues in assessing antibiotic use in a low-resource setting, including the lack of large databases, such as those used in high-income countries. The reported rates of antibiotic utilization in LMICs are challenging to compare with those from high-income countries, due to different data collection and reporting methods, variation in antibiotic prescribing practices, and drug availability and use. It is difficult to define what would constitute an adequate rate of antibiotic use in a specific population [8]. Many factors must be considered when discussing the desirable levels of antibiotic consumption in a certain country, including local disease prevalence, antibiotic prescribing practices, and the philosophy of their use [9]. Comparisons with other, similar populations helps to give context; therefore, reports on the practices of antibiotic use in low-resource settings are essential to help set a baseline and develop effective interventions towards rational drug prescribing when appropriate. 

In Syria, a disastrous war has affected health care provision, and the burden of infections, morbidity, and mortality continues to grow [10]. The gross domestic product (GDP) loss due to AMR is estimated to be between USD 2 billion and USD 159 billion per year over 40 years in the Middle East and North Africa (MENA) (including Syria) [11]. The irrational use of antibiotics is widespread, and this public health concern extends far beyond Syria to other Middle Eastern neighboring countries [12]. Antibiotics are classified as prescription-only medication in Syria, requiring a physician’s prescription to be dispensed [13], but community pharmacists commonly dispense antibiotics over the counter [12]. Antibiotic resistance, resulting from erroneous prescribing and overprescribing antibiotics, is widespread in Syria [10]. To tackle this problem, gathering reliable data on the patterns of antibiotic use is the necessary first step.

A recent study that described outpatient antibiotic utilization in Syria found evidence of the misuse of antibiotics. This included high rates of broad-spectrum antibiotic dispensing and a high proportion of the WHO Watch group of antibiotics [14]. Substantial use of fixed-dose combinations of broad-spectrum antibiotics was also reported [14]. According to the WHO, the use of fixed-dose combinations of multiple broad-spectrum antibiotics is not evidence-based, and is not recommended in clinical practice [15,16]. The proneness to prescribing fixed-dose antibiotic combinations could imply that prescribing multiple antibiotics within a single treatment could also be common in the Syrian setting. To the best of our knowledge, no studies explicitly focused on the patterns of combination antibiotic use in outpatients. If widespread and unnecessary, this practice can significantly contribute to AMR, but without increased knowledge on this subject matter, these findings may remain hidden. Therefore, this follow-up study was designed to fill this knowledge gap by determining the prevalence and prescribing patterns of combination antibiotic dispensing in Syria.

## 2. Results

Out of 59,404 prescriptions for antibiotics, there were 8899 (14.98%) that contained more than one different antibiotic. The rate of total antibiotic prescribing was 730.55 and the combination prescription rate was 109.44 prescriptions per 1000 persons per year. The majority of prescriptions (8770, 98.5%) contained two different antibiotics, while a lesser number contained three (128, 1.44%) or four (1, 0.06%) different agents. Out of 33,444 patients prescribed antibiotics, 7524 (22.49%) received a combination of more than one agent. The age and sex stratification of patients dispensed antibiotic combination therapy is presented in Table 1. In females, the largest number of patients were between 30 and 49 years old, compared to 40 to 59 years old in males. Out of 7524 patients, the majority received prescriptions containing antibiotic combinations once (6374, 84.70%) or twice (961, 12.77%) +, but some patients were prescribed antibiotic combinations three (159, 2.11%) or four (27, 0.36%) times during the study period. A negligible number of patients were prescribed an antibiotic combination five or more times (3, 0.04%). 

**Figure 1:**
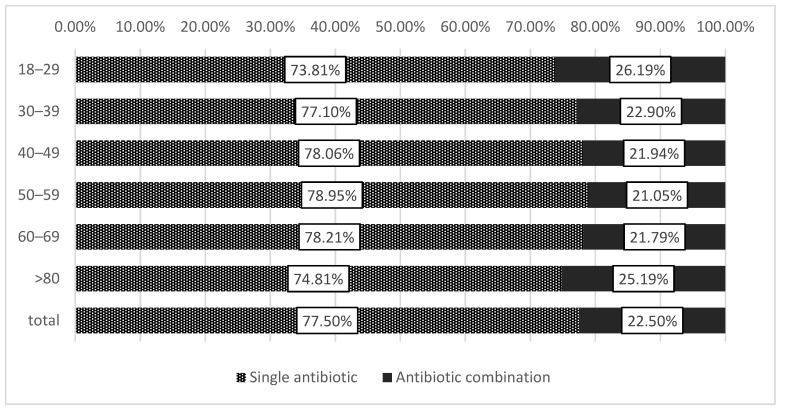
Prevalence of dispensing combination antibiotic therapy in patients of different age groups (combination antibiotic therapy defined as receiving a prescription for two or more different antibiotics during one visit).

**Figure 2:**
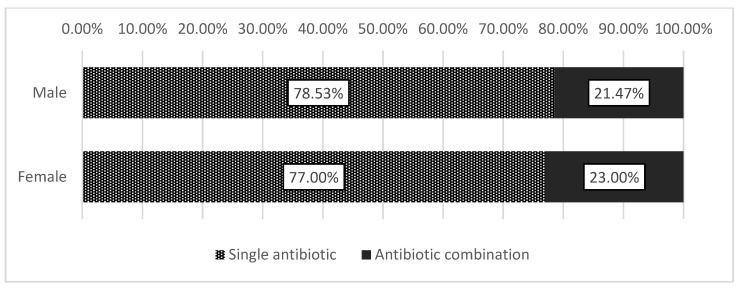
Prevalence of dispensing combination antibiotic therapy in patients of different sex groups during one year (combination antibiotic therapy defined as receiving a prescription for two or more different antibiotics during one visit).

Based on the number of patients, the overall prevalence of antibiotic combination therapy was 22.49%. The dispensing of an antibiotic combination was most common in the youngest (18–30 years, 26.19%) and the oldest age groups (>70 years, 25.19%), *p* < 0.001 (Figure 1). As shown in Figure 2, a larger share of female patients were dispensed antibiotic combinations compared to men (23.00% vs. 21.47%, *p* < 0.001). Additionally, the sex-standardized rates of dispensed antibiotic combinations, expressed in packages of antibiotics per 1000 persons per year, were 119.56 in female vs. 92.73 in male beneficiaries.

The top ten most common antibiotic combinations dispensed, accounting for 77.82% of the total dispensing of combination antibiotics, are presented in Table 2. According to the pharmacological subgroups (ATC4), the combination of penicillins, including beta-lactamase inhibitors and third-generation cephalosporins, was the most commonly dispensed, accounting for 27.27% of all prescriptions, followed by the combination of third-generation cephalosporins and macrolides, accounting for 12.28% for macrolides, 12.10% for third-generation cephalosporins and 9.35% for second-generation and third-generation cephalosporins). 

Table 3 presents the age-standardized rates of dispensing for the ten most common antibiotic combinations. According to the pharmacological subgroups (ATC4), combinations containing cephalosporins with macrolides and fluoroquinolones were more commonly used in patients over 60 years old. For the combination of third-generation cephalosporins with fluoroquinolones, the age-standardized rates, expressed as the number of prescriptions per 1000 persons per year, were between 7.67 and 13.37 in patients aged 60–69 and over 70, respectively, which are much higher than for patients in other age groups. Dual therapy with cephalosporins was the most common in patients younger than 40 (*p* < 0.001), with the highest age-standardized rates reported in the youngest age group (18–29 years, 17.25 to 20.57 prescriptions per 1000 persons per year). Combinations of penicillins with BLI and lincosamides were the most common in the 40–49 age group.

When looking at individual agents, the top ten combinations, accounting for more than 50% of total combination antibiotic utilization, are presented in Table 4. The most commonly dispensed combination was amoxicillin (alone or in combination with clavulanic acid) and ceftriaxone (alone or in combination with sulbactam), accounting for more than a quarter of all prescriptions. Additionally, 67.00% of the prescriptions contained a combination of antibiotics including ceftriaxone, alone or in combination with sulbactam (Supplementary Table S1). Combinations of amoxicillin and BLI with ceftriaxone, amoxicillin and BLI with ceftriaxone and BLI, and ceftriaxone and BLI with clarithromycin ranked first, second and third, respectively, among both females and males. The combination of amoxicillin and BLI with lincomycin was used more commonly in males than in females, while the combination of ceftriaxone and cefdinir ranked seventh among females, and did not make it into the top ten most commonly used antibiotic combinations in male patients. 

## 3. Discussion

The present study has brought light to the high rate and worrying pattern of the dispensing of combinations of broad-spectrum antibiotics in outpatients with health insurance in Syria. 

Almost a quarter (22.49%) of all patients receiving antibiotics in the outpatient setting received an antibiotic combination at least once during 12 months. Out of all the prescriptions for antibiotics, 14.98% contained more than one antibiotic. Combinations of antibiotics increase the selection of resistance through the exposure of bacteria to these drugs, leading to increasing costs and chances of side effects, resulting from drug interactions [17]. The unnecessary use of antimicrobials, especially those with a broad spectrum, should be avoided [18]. Ten recommendations to physicians prescribing antibiotics to outpatients, compiled by a multinational working group from the International Society of Chemotherapy, suggest limiting the dispensing of antibiotic combinations in outpatients to only specific situations, such as infections caused by *H. pylori* and tuberculosis [17]. Moderate-to-severe community-acquired pneumonia of unknown etiology in outpatients can be managed by combining beta-lactams and macrolides before resorting to fluoroquinolones [19]. Unfortunately, there are few data on the frequency of antibiotic combination therapy in outpatients. Studies from LMICs rarely used datasets that would allow for the identification of multiple-antibiotic dispensing. However, the rates reported in the available literature are much lower than those identified in the present study. In a study from Hungary, based on the patient-level dispensing data from the Hungarian National Health Fund Administration database, combination therapy was prescribed for only 3% of the exposed inhabitants [20]. In Ethiopia, a study that measured the volume of antibiotic consumption in the outpatient departments of a tertiary-care teaching hospital reported that 16.53% of the patients received a combination of antibiotics [21]. Taking all of the above into account, the rate of prescribing combination antibiotics in Syria seems excessive. 

There are several possible reasons for why a physician may prescribe combinations of broad-spectrum antibiotics in the Syrian setting. A review article on the causes of antimicrobial resistance in LMIC countries identified a fear of bad treatment outcomes as one reason why physicians blindly prescribe multiple and broad-spectrum antimicrobials [22]. Adequate infrastructure for diagnostics is a prerequisite for the timely diagnosis and adequate selection of suitable antibiotic therapy. In Syria, both governmental and non-governmental areas are struggling with a lack of trained staff who are competent in diagnosing and managing infections, particularly those caused by resistant organisms [23]. The lack of confidence in the quality of available medicines could also drive doctors to prescribe multiple antibiotics. There is an issue with the availability of medicines in line with the international quality control standards in Syria, stemming from the 2016 ban on importing all medicines that could be purchased locally [24]. As a result, patients are dependent on locally manufactured medicines of uncertain quality. A study that aimed to identify problems related to the quality of locally manufactured pharmaceutical dosage forms in Syria confirmed that the circumstances of the war led to the lack of control over pharmaceutical dosage forms, and led to the poor quality of locally manufactured medications [25]. Poor-quality antibiotics can be one of the reasons to use more aggressive antimicrobial treatment regimens [26]. Studies analyzing the factors influencing the rate of prescribing antibiotic combinations are scarce. A recent study from China used the prescription rate of antibiotics and the prescription rate of antibiotic combinations as a proxy for the rationality of physicians’ antibiotic prescriptions. The findings imply that knowledge was a strong predictor of rational antibiotic prescribing. Physicians with a higher level of knowledge of antibiotics prescribed antibiotic combinations less often [27].

The prevalence of dispensing antibiotic combinations was higher in the youngest (18–30 years) and oldest (>70 years) age groups. Higher antibiotic use in the elderly is in line with the available evidence. In a systematic review where nineteen studies exploring age as a factor in antibiotic use were included, thirteen found a statistically significant association between older age and higher odds of antibiotic prescription [28]. A study from the US identified older adults (≥65 years) as having the highest outpatient antibiotic prescribing rate of all age groups [29]. The high rate of antibiotic combinations in the youngest age group in Syria could result from younger people being the most likely to be involved in the conflict and exposed to conflict-related injuries [14]. A larger share of females, in comparison with male patients, were prescribed antibiotic combinations. Previous studies also reported on females using more antibiotics than males, which different incidences of certain infections could explain, but these findings could also point towards possible over-prescription to females [30,31,32].

The most commonly used combinations were those of penicillins, including beta-lactamase inhibitors and third-generation cephalosporins, followed by the combination of third-generation cephalosporins and macrolides, and two different cephalosporins. Combinations containing cephalosporins with macrolides and fluoroquinolones were more commonly used in patients over 60 years of age, while dual therapy with cephalosporins was more common in patients under 40 years of age. Combinations of beta-lactams with macrolides and fluoroquinolones are guideline-concordant therapies for moderate-to-severe community-acquired pneumonia. The problem of dual beta-lactam therapy was obvious when analyzing individual agents; the most commonly dispensed combination was amoxicillin, alone or in combination with clavulanic acid, together with ceftriaxone, or a ceftriaxone–sulbactam combination. The purpose of antibiotic combinations is to expand the spectrum of activity and enhance the effectiveness of the treatment. Antibiotic combinations should aim for a synergistic effect, while avoiding antagonistic and incompatible effects. The benefits of dual beta-lactam therapy remain unclear in the outpatient setting. Dual beta-lactam therapy is a strategy reserved for serious Gram-negative infections, as combinations of carbapenems were used in clinical trials as a rescue strategy for multidrug-resistant bacteria. Therefore, the dispensing of combinations of beta-lactams identified in the present study seems unwarranted. Worryingly, a large share of the identified antibiotic combinations (more than 60% of prescriptions) contained ceftriaxone, alone or in combination with sulbactam. Other studies have shown that ceftriaxone is the most common parenteral antibiotic in outpatient settings [14,33,34]. The efficacy against a broad range of microorganisms, pharmacokinetics allowing for a convenient once-daily administration, and a generally excellent safety profile are among the factors that make it suitable for use in the outpatient setting [35]. A fixed-dose combination of ceftriaxone and sulbactam is one of the examples of drugs not recommended by the WHO because it is not evidence-based nor recommended in high-quality international guidelines [16]. The high use of ceftriaxone in antibiotic combination therapy in outpatients adds to the evidence of the misuse of antibiotics in Syria [14]. The consequences of broad-spectrum antibiotic overprescribing pose a huge threat to global health, but can also result in a tangible health impact for these patients.

The present study had limitations that need to be mentioned. First, obtaining antibiotics without a prescription is common practice in Syria, but the data in the present study only included antibiotics prescribed by physicians. The results are representative of a population in Syria with health insurance, but are not generalizable to the whole population. The data did not include the drugs prescribed to treat diseases not included in the health insurance coverage in Syria (treatment for sexually transmitted diseases or dental conditions). Standard measures of consumption, such as defined daily dose and defined daily dose per 1000 inhabitants per day, were not used in the analysis, making a direct comparison with other studies difficult. Finally, the lack of data on diagnosis did not allow for further analysis of the indications for which antibiotic combinations were dispensed. Still, this study contributes to our knowledge about antibiotic prescribing and misuse in LMICs. Considering the large sample size, the use of standardized methods for the drug classification and analysis, and the paucity of studies from this region of the world, the present study provides an important addition to the current knowledge on an often under-reported issue in antibiotic use.

## 4. Materials and Methods

### 4.1. Setting and Data Source

This retrospective cross-sectional study was based on the outpatient dispensing data from thirteen Syrian governates, excluding the Ar-Raqqa governorate, which is not under Syrian government control. The health insurance data from June 2018 to May 2019 containing outpatient medication dispensing were used. Data included the proprietary drug name, number of dispensed packages, pharmaceutical formulation and route of drug administration, prescription number, a unique number for each patient, date of dispensing, the name of the governorate, and patient’s age and sex. Unique prescription numbers allowed for analysis on the exact pattern of dispensed antibiotics during a single dispensing event. Data for 81,314 beneficiaries were present in the dataset. A detailed description of the study population is available in the previous publication [14]. 

### 4.2. Data and Statistical Analysis

The original dataset contained all dispensed medicines for the study population. Only medicines belonging to the ATC group J01 (antibacterials for systemic use) were included in the analysis. The 2020 version of the World Health Organization (WHO) ATC/DDD index was used to assign ATC codes to the respective international non-proprietary names (INN) [36]. The combination use was analyzed according to the third, fourth and fifth levels of the ATC classification. The dispensing of antibiotic combinations was defined as dispensing more than one different antibiotic to a single patient during a single dispensing event. Prescriptions containing multiple packages of a single antibiotic were carefully selected and excluded from the analysis on the combination antibiotic therapy. Patients were classified as receiving antibiotic combination therapy if they received a prescription containing more than one antibiotic at least once during the study period. Results were reported by the number of patients, the number of prescriptions, and the age and sex-adjusted rates expressed as number of prescriptions per 1000 persons per year. All statistical assessments were performed using Excel 2019 and IBM SPSS Statistics version 25 (SPSS Inc., Chicago, IL, USA). The chi-squared test was used to compare the prevalence of antibiotic combination dispensing according to age and sex. A *p*-value less than 0.05 was considered significant. 4.3. Ethical Considerations

Each patient was given a unique identification number, but individual patients could not be identified. After the assessment, the ethics commission of the University of Bayreuth stated that their approval was not required for the analysis and reporting of the dispensing data in the present study.

## 5. Conclusions

The present study gives evidence of a serious and previously unreported issue of using broad-spectrum antibiotics in combination in Syrian outpatients. This practice, far from appropriate drug use, poses a risk to local and global health by promoting AMR, and puts patients at an increased risk of side effects. Further studies on the exact reasons for the widespread prescribing of combination antibiotics by Syrian doctors are warranted. Nevertheless, the present study contributes to the knowledge on the role of antibiotic combination therapy in LMICs, a problem that must not be overlooked in future studies and during the development of antibiotic stewardship policies.

## Figures and Tables

**Table 1:** Number and share of patients who were prescribed more than one antibiotic during a single visit according to the patients’ age and sex.

Age Category	Female		Male		Total	
	*n*	%	*n*	%	*n*	%
18–29	805	15.63%	121	5.09%	926	12.31%
30–39	1614	31.35%	488	20.55%	2102	27.94%
40–49	1256	24.39%	738	31.07%	1994	26.50%
50–59	1082	21.01%	623	26.23%	1705	22.66%
60–69	337	6.54%	266	11.20%	603	8.01%
≥70	55	1.07%	139	5.85%	194	2.58%
Total	5149	100.00%	2375	100.00%	7524	100.00%

**Table 2:** Top ten dispensed antibiotic combinations ranked by the share of total dispensing by pharmacological subgroups (ATC4) expressed as the number of prescriptions.

Rank	ATC (INN)	No (%)	Rate **
1.	J01CR + J01DD	2427 (27.27%)	29.85
2.	J01DD + J01FA	1093 (12.28%)	13.44
3.	J01DD + J01DD	1077 (12.10%)	13.24
4.	J01DC + J01DD	832 (9.35%)	10.23
5.	J01DD + J01MA	576 (6.47%)	7.08
6.	J01CR + J01FF	239 (2.69%)	2.94
7.	J01DD + J01GB	195 (2.19%)	2.40
8.	J01CA + J01FA	195 (2.19%)	2.40
9.	J01CR + J01MA	154 (1.73%)	1.89
10.	J01CR + J01FA	137 (1.54%)	1.68
Top 10		6925 (77.82%)	85.16
Other (11–144)		1974 (22.18%)	24.28
Total		8899 (100.00%)	109.44

** No. of prescriptions per 1000 persons/year. Abbreviations: J01CR + J01DD (combinations of penicillins, including BLI* + 3rd-generation cephalosporins), J01DD + J01FA (3rd-generation cephalosporins + macrolides), J01DD + J01DD (3rd-generation cephalosporins + 3rd-generation cephalosporins), J01DC + J01DD (2nd-generation cephalosporins + 3rd-generation cephalosporins), J01DD + J01MA (3rd-generation Cephalosporins + fluoroquinolones), J01CR + J01FF (combinations of penicillins, including BLI + lincosamides), J01DD + J01GB (3rd-generation cephalosporins + other aminoglycosides), J01CA + J01FA (penicillins with extended spectrum + macrolides), J01CR + J01MA (combinations of penicillins, including BLI + fluoroquinolones), J01CR + J01FA (combinations of penicillins, including BLI + macrolides). BLI—beta-lactamase inhibitors.

**Table 3:** Age-standardized rates of use for ten most common antibiotic combinations according to the pharmacological subgroup in different age groups in number of prescriptions per 1000 persons per year.

ATC	Dispensing Rate					
	Age Group					
	18–29	30–39	40–49	50–59	60–69	≥70
J01CR + J01DD	36.22	40.04	31.06	25.44	15.53	27.46
J01DD + J01FA	15.03	16.70	13.50	11.17	11.65	13.73
J01DD + J01DD	20.57	18.95	13.69	8.98	6.87	9.75
J01DC + J01DD	17.25	13.50	11.85	7.52	3.29	3.97
J01DD + J01MA	5.54	8.49	5.90	6.89	7.67	13.37
J01CR + J01FF	3.82	3.94	3.53	2.60	0.80	0.00
J01DD + J01GB	3.33	2.87	2.42	1.87	2.39	0.72
J01CA + J01FA	2.09	2.81	2.37	2.01	2.89	2.17
J01CR + J01MA	1.85	1.35	2.52	1.69	1.69	3.97
J01CR + J01FA	2.96	2.25	1.94	0.78	1.49	1.08

Abbreviations: J01CR + J01DD (combinations of penicillins, including beta-lactamase inhibitors (BLI) + 3rd-generation cephalosporins), J01DD + J01FA (3rd-generation cephalosporins + macrolides), J01DD + J01DD (3rd-generation cephalosporins + 3rd-generation cephalosporins), J01DC + J01DD (2nd-generation cephalosporins + 3rd-generation cephalosporins), J01DD + J01MA (3rd-generation cephalosporins + fluoroquinolones), J01CR + J01FF (combinations of penicillins, including BLI + lincosamides), J01DD + J01GB (3rd-generation cephalosporins + other aminoglycosides), J01CA + J01FA (penicillins with extended spectrum + macrolides), J01CR + J01MA (combinations of penicillins, including BLI + fluoroquinolones), J01CR + J01FA (combinations of penicillins, including BLI + macrolides). BLI—beta-lactamase inhibitors.

**Table 4:** Top ten dispensed antibiotic combinations ranked by the share of total dispensing by the individual agents (ATC5), expressed as the number of prescriptions and the number of prescriptions per 1000 persons per year.

Rank	ATC (INN)	No (%)	Rate *
1.	J01CR02 + J01DD04 (amoxicillin and BLI* + ceftriaxone)	1567 (17.61%)	19.27
2.	J01CR02 + J01DD63 (amoxicillin and BLI + ceftriaxone and BLI)	723 (8.12%)	8.89
3.	J01DD63 + J01FA09 (ceftriaxone and BLI + clarithromycin)	391 (4.39%)	4.81
4.	J01DD04 + J01DD08 (ceftriaxone + cefixime)	336 (3.78%)	4.13
5.	J01DD04 + J01FA09 (ceftriaxone + clarithromycin)	330 (3.71%)	4.06
6.	J01DC02 + J01DD04 (cefuroxime + ceftriaxone)	298 (3.35%)	3.66
7.	J01DD04 + J01DD15 (ceftriaxone + cefdinir)	235 (2.64%)	2.89
8.	J01DD08 + J01DD63 (cefixime + ceftriaxone and BLI)	231 (2.60%)	2.84
9.	J01DC02 + J01DD63 (cefuroxime + ceftriaxone and BLI)	226 (2.54%)	2.78
10.	J01CR02 + J01FF02 (amoxicillin and BLI + lincomycin)	218 (2.45%)	2.68
Top 10		4555 (51.19%)	56.02
Other (11–402)		3455 (38.82%)	42.49
Total		8899 (100.00%)	109.44

* No of prescriptions per 1000 persons per year. Abbreviations: J01CR02 + J01DD04 (amoxicillin and BLI* + ceftriaxone), J01CR02 + J01DD63 (amoxicillin and BLI + ceftriaxone and BLI), J01DD63 + J01FA09 (ceftriaxone and BLI + clarithromycin), J01DD04 + J01DD08 (ceftriaxone + cefixime), J01DD04 + J01FA09 (ceftriaxone + clarithromycin), J01DC02 + J01DD04 (cefuroxime + ceftriaxone), J01DD04 + J01DD15 (ceftriaxone + cefdinir), J01DD08 + J01DD63 (cefixime + ceftriaxone and BLI), J01DC02 + J01DD63 (cefuroxime + ceftriaxone and BLI), J01CR02 + J01FF02 (amoxicillin and BLI + lincomycin).

## Data Availability

Data used in the analysis are available from the authors upon reasonable request.

## Supplementary Materials

The following are available online at https://www.mdpi.com/article/10.3390/antibiotics11010074/s1: Table S1. top 10 antibiotic combinations in patients of different sex according to the number of prescriptions and number of prescriptions per 1000 persons per year.
