# Supplementary material for: The Overlooked Issue of Outpatient Combination Antibiotic Prescribing in Low- and Middle-Income Countries: An Example from Syria
Source: Antibiotics (Basel). 2022 Jan 9;11(1):74. doi: 10.3390/antibiotics11010074 (PMC8772973; doi:10.3390/antibiotics11010074)
Supplement: Supplementary file 1 [file antibiotics-11-00074-s001.zip › antibiotics-1522270-supplementary.pdf]

antibiotics

Table S1. Top 10 antibiotic combinations in patients of different sex according to the number of prescriptions and number of prescriptions per 1000 persons per year.

Sex	ATC (INN)	No (%)	Rate*
Female	J01CR02+J01DD04 (Coamoxiclav+Ceftriaxone)	981(16.20%)	19.37
	J01CR02+J01DD63 (Amoxicillin and BLI+Ceftriaxone and BLI)	486(8.03%)	9.60
	J01DD63+J01FA09 (Ceftriaxone and BLI+Clarithromycin)	255(4.21%)	5.04
	J01DD04+J01DD08 (Ceftriaxone+Cefixime)	233(3.85%)	4.60
	J01DC02+J01DD04 (Cefuroxime+Ceftriaxone)	230(3.80%)	4.54
	J01DD04+J01FA09 (Ceftriaxone+Clarithromycin)	214(3.53%)	4.23
	J01DD04+J01DD15 (Ceftriaxone+Cefdinir)	185(3.06%)	3.65
	J01DD08+J01DD63 (Cefixime+Ceftriaxone and BLI)	162(2.68%)	3.20
	J01DC02+J01DD63 (Cefuroxime+Ceftriaxone and BLI)	162(2.68%)	3.20
	J01CR02+J01FF02 (Amoxicillin and BLI+Lincomycin)	136(2.25%)	2.69
	Top 10 Total	3044(50.27%)	60.11
	Other	3011(49.73%)	59.46
	Total Female	6055(100.00%)	119.56
Male	J01CR02+J01DD04 (Amoxicillin and BLI+Ceftriaxone)	586(20.60%)	19.11
	J01CR02+J01DD63 (Amoxicillin and BLI+Ceftriaxone and BLI)	237(8.33%)	7.73
	J01DD63+J01FA09 (Ceftriaxone and BLI+Clarithromycin)	136(4.78%)	4.43
	J01DD04+J01FA09 (Ceftriaxone+Clarithromycin)	116(4.08%)	3.78
	J01DD04+J01DD08 (Ceftriaxone+Cefixime)	103(3.62%)	3.36
	J01CR02+J01FF02 (Amoxicillin and BLI+Lincomycin)	82(2.88%)	2.67
	J01DD04+J01FA10 (Ceftriaxone+Azithromycin)	79(2.78%)	2.58
	J01DD08+J01DD63 (Cefixime+Ceftriaxone and BLI)	69(2.43%)	2.25
	J01CA04+J01FA09 (Amoxicillin+Clarithromycin)	68(2.39%)	2.22
	J01DC02+J01DD04 (Cefuroxime+Ceftriaxone)	68(2.39%)	2.22
	Top 10 Yotal	1544(54.29%)	50.34
	Other	1300(45.71%)	42.39
	Total Male	2844(100.00%)	92.73

*No of prescriptions per 1000 persons/ year.
